# Recent Advances in Chitosan-Based Carriers for Gene Delivery

**DOI:** 10.3390/md17060381

**Published:** 2019-06-25

**Authors:** Ye Cao, Yang Fei Tan, Yee Shan Wong, Melvin Wen Jie Liew, Subbu Venkatraman

**Affiliations:** School of Materials Science & Engineering, Nanyang Technological University, Singapore 639798, Singapore; ycao@ntu.edu.sg (Y.C.); yftan@ntu.edu.sg (Y.F.T.); YSWong@ntu.edu.sg (Y.S.W.); LIEW0182@e.ntu.edu.sg (M.W.J.L.)

**Keywords:** chitosan, siRNA, DNA, gene delivery, nanoparticles, polyplex, complex, nucleic acid delivery, sustained- release, gene knock-down, gene therapy

## Abstract

Approximately 4000 diseases are associated with malfunctioning genes in a particular cell type. Gene-based therapy provides a platform to modify the disease-causing genes expression at the cellular level to treat pathological conditions. However, gene delivery is challenging as these therapeutic genes need to overcome several physiological and intracellular barriers in order, to reach the target cells. Over the years, efforts have been dedicated to develop efficient gene delivery vectors to overcome these systemic barriers. Chitosan, a versatile polysaccharide, is an attractive non-viral vector material for gene delivery mainly due to its cationic nature, biodegradability and biocompatibility. The present review discusses the design factors that are critical for efficient gene delivery/transfection and highlights the recent progress of gene therapy using chitosan-based carriers.

## 1. Introduction

Gene therapy has grown very rapidly because of its tremendous therapeutic potential to cure numerous genetic diseases by the insertion of new genes (DNA and RNA) into target cells with expression of the transgene. However, the in vivo direct delivery of naked therapeutic genes is unrealistic and fraught with challenges, including the susceptibility of gene degradation by nucleases in the plasma, non-specificity towards targeted cells, as well as the inability of the negatively charged genes from entering negatively charged cellular membranes. Indeed, with almost 2600 gene therapeutics having completed or undergoing clinical trials, only six gene therapeutics have received approval in the west [1,2]. It is now recognized that the administration of naked genes either systemically or locally is not efficacious, due to the issues mentioned above. Therefore, the basic challenge for gene therapy is the development of safe and efficient gene transfection vectors. 

Researchers have explored several different vectors, which are categorized into two groups: viral and non-viral. Although viral vectors are involved in two-thirds of the trials [2], non-viral methods are attracting increased attention. While viral vectors have high transfection efficiency and high levels of gene expression, their major disadvantages include unwanted immune responses, possible toxicity, potential immunogenicity, low loading capacity and fatal inflammation [3,4]. Thus, there is a need to have good alternatives to viral vectors for gene delivery, including delivery of gene silencing molecules.

Non-viral vectors have a superior safety profile with low immune response, the possibility of facile large-scale production, high loading efficiency for bioactives, and unrestricted gene materials size [5]. However, their ability to enter cells and gene transfection efficiencies are lower than that of viral vectors. Non-viral vectors can be categorized into two groups: cationic lipids (liposomes and lipoplexes) and polymers (polyplexes) [6,7,8], which include liposomes, complexes of positively charged polymers and negatively charged genes, and nanoparticles [9]. Several liposomes and lipoplexes (complexes of gene and cationic lipids) have been widely studied. However, liposomes usually have a low encapsulation efficiency and no sustained release. Cationic lipids, such as DOTAP, (1,2-dioleoyl-3-trimethylammonium-propane) and DOTMA (N-[1-(2,3-dioleoyloxy) propyl]-N,N,N-trimethyl-ammonium methyl sulphate) can form lipoplexes with negatively charged genes to form nanoparticles by electrostatic interaction, providing high in vitro transfection efficiency [3]. 

Although lipoplexes offer several advantages as gene delivery carriers, their potential dose dependent-toxicity (from positively charged head) and short life spans should be carefully investigated before in vivo studies or clinical trials [10]. 17.7% of gene therapy clinical trials use naked DNA because it offers a much safer option than viral vectors. However, this approach lacks target specificity, shows low transfection efficiency and fast degradation. Lipofection was the second most popular non-viral vector and is used in 4.5% of clinical trials worldwide, with cationic lipid/DNA complexes [11]. However, no long-term knock down effect (sustained-release) in cells has been reported for cationic lipids-based gene delivery systems, which means that continuous infusion or frequent repeat administrations are necessary. 

Cationic polymers have been widely investigated as gene delivery systems, mainly because they inherently offer unlimited gene packaging capacities and allow extensive modifications [12]. Among all the cationic polymers, polyethyleneimine (PEI) is the most studied cationic polymer for gene delivery due to its strong gene complexation and high transfection efficiency. However, its application is limited in clinical trials because of its substantial toxicity issue [7,13].

To address the toxicity issue, chitosan-based carriers have gained increasing interest as a safe delivery system for gene materials including plasmid DNA (pDNA), oligonucleotides and siRNA. Unlike other cationic polymers, chitosan has several advantages such as low toxicity (LD_50_:16 g/kg, while LD_50_ for NaCl is 3 g/kg) [14], low immunogenicity, excellent biocompatibility as well as a high positive charge. Similarly, chitosan can form complexes with negatively charged genes easily due to its abundant amine groups. However, clinical translation of chitosan-based gene delivery carriers is still unsatisfactory due to several challenges like poor water solubility, charge deduction at physiological pH and poor targeting capability. In this review paper, we summarise current progress of chitosan-based formulations for gene delivery, discuss advantages and disadvantages of latest chitosan nanoparticle formulations and introduce the emergence of a new generation of gene delivery carriers.

## 2. Design Criteria and Requirements for Gene Delivery Using Chitosan-Based Carriers

### 2.1. Design Considerations for Chitosan-Based Carriers

In RNA interference (RNAi) therapy, double-stranded small interfering RNAs (siRNAs) assemble into endoribonuclease associates to produce RNA-induced silencing complex (RISC). RNA strands guide the RISCs to complementary RNA molecules and then target the specific sequence in a messenger RNA (mRNA), leading to mRNA cleavage or degradation and finally inhibit the synthesis of protein. The specificity in regulating specific proteins makes RNAi technology an attractive choice for future therapeutics. However, the clinical utility of systemically-administered therapeutic siRNA remains a challenge due to the nature of the siRNA. Naked RNA is sensitive to nucleases and has a very short half-life. In addition, the cell-penetrating ability of naked siRNA is quite poor. Thus, an efficient, safe and effective carrier is mandated during the transportation to targeted diseased cells. 

DNA therapy faces similar challenges, with the additional requirement to enter the nucleus of a target cell. An ideal polymeric delivery system must have the following properties: be able to form a stable complex/nanoparticle upon coupling with nucleic acids, protect the cargo from nuclease degradation, and target the desired cells and promote cellular entry. Once inside the cell, the carrier and cargo must escape from endosomes and lysosomes, and then release (by decomplexation or unpacking) genes in the cytoplasm, interact with target cellular elements while showing low toxicity [12]. Chitosan has been widely studied as a gene delivery carrier due to its unique advantages, such as its cationic nature, biocompatibility, relatively low cost of production, and facile functional modification. Due to the versatility of chitosan, several factors must be considered while selecting the optimal chitosan for efficient carrier action, such as chitosan molecular weight [15], chitosan derivatives, chitosan deacetylation degree, and N/P ratio.

### 2.2. Basic Properties of Chitosan

**Figure 1:**
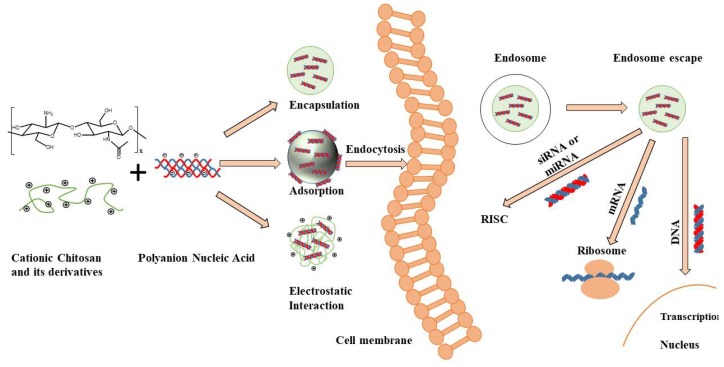
Chitosan structure, fabrication mechanisms of chitosan-nucleic acid-based particles and transection mechanisms for different nucleic acids. Positively charged Chitosan has the ability to interact with negatively charged nucleic acids resulting in the spontaneous formation of nanoparticles/polyplexes. After cellular uptake by endocytosis, these vectors need to escape from endosomes and release (de-complex) into the cytoplasm. The released siRNA and microRNA (mRNA) must bind to the RNA-induced silencing complex (RISC) to activate the RNA interfering pathway, while messenger RNA (mRNA) must be loaded into the translation machinery. DNA must be further transported into the nucleus to work [7].

Chitosan, extracted from the exoskeleton of shellfish (crabs, shrimps, etc.), offers the advantage of being biodegradable and biocompatible, characteristics that are highly desired in a drug delivery system. Chitosan is a polysaccharide consisting of repeating D-glucosamine and N-acetyl-d-glucosamine units. After deacetylation, each subunit of chitosan contains a primary amine group (pKa = 6.5) and two hydroxyl groups, which can be easily chemically modified according to the application. At pH < 6.5, the amine groups of chitosan protonate to NH_3_^+^ and chitosan becomes soluble and cationic. In addition to pH, the solubility of chitosan is greatly influenced by the degree of deacetylation (DD), and molecular weight [15,16]. Highly positively charged chitosan can complex with genetic material easily and efficiently, making chitosan an attractive candidate for gene delivery (Figure 1). Chitosan properties must be understood and optimized in order to achieve efficient and affective gene silencing, including (i) chitosan molecular weight, (ii) chitosan derivatives properties, (iii) deacetylation degree and (iv) N/P ratio.

### 2.3. Chitosan Molecular Weight

To protect genetic material for cellular internalization, chitosan particles or complexes need to have appropriate stabilities that not only effectively protect genes, but also permit gene release inside the cell after uptake. The MW of chitosan can affect the size and stability of particles or complexes, cellular uptake and release of gene inside cytoplasm, and finally influences transfection efficiency. Firstly, chitosan MW affects the formation of nanoparticles or polyplexes. For example, 2 kDa MW chitosan cannot form stable nanoparticles, while previous studies proved that 25~50 kDa chitosan could form stable nanoparticles with siRNA and offer better protection against enzyme degradation than <10 kDa MW chitosan. According to literature, only chitosan molecules that are 5–10 times larger than siRNA will form stable nanoparticles within the size range of 100–300 nm [17]. Therefore, the optimal MW range of chitosan to form nanoparticles with siRNA should be between 65~170 kDa [18]. 

Secondly, different MW chitosan will form different sized nanoparticles. For example, 20, 40 and 80 kDa chitosan can formed 200, 250 and 300 nm nanoparticles, respectively [19]. McLaughlin et al. [20] reported how chitosan MW influenced the size of chitosan /plasmid complexes. Similarly, Huang et al. [21] found that the mean particle size increased from 155 to 181 nm when increasing the chitosan MW from 48 to 213 kDa. Thirdly, the MW of chitosan indirectly influences the transfection efficiency. In one such study, 55 kD chitosan showed higher transfection efficiency than 16 and 35 kD chitosans [22]. According to literature, high MW chitosans are preferred over the lower ones due to enhanced stability and better protection in the endosomal/lysosomal compartments. However, high MW chitosans will restrict the release of genes and in turn delay transfection because of strong binding between chitosan and gene molecules. On the other hand, Sato et al. [23] reported that 100 kDa chitosan was less efficient than 15 and 52 kDa chitosans for transfection in A549 cells, B16 melanoma cells, and Hela cells. In summary, an appropriate MW of chitosan should be carefully selected to achieve desired stability, gene release rate and protection, and transfection efficiency.

### 2.4. Chitosan Derivatives

**Figure 2:**
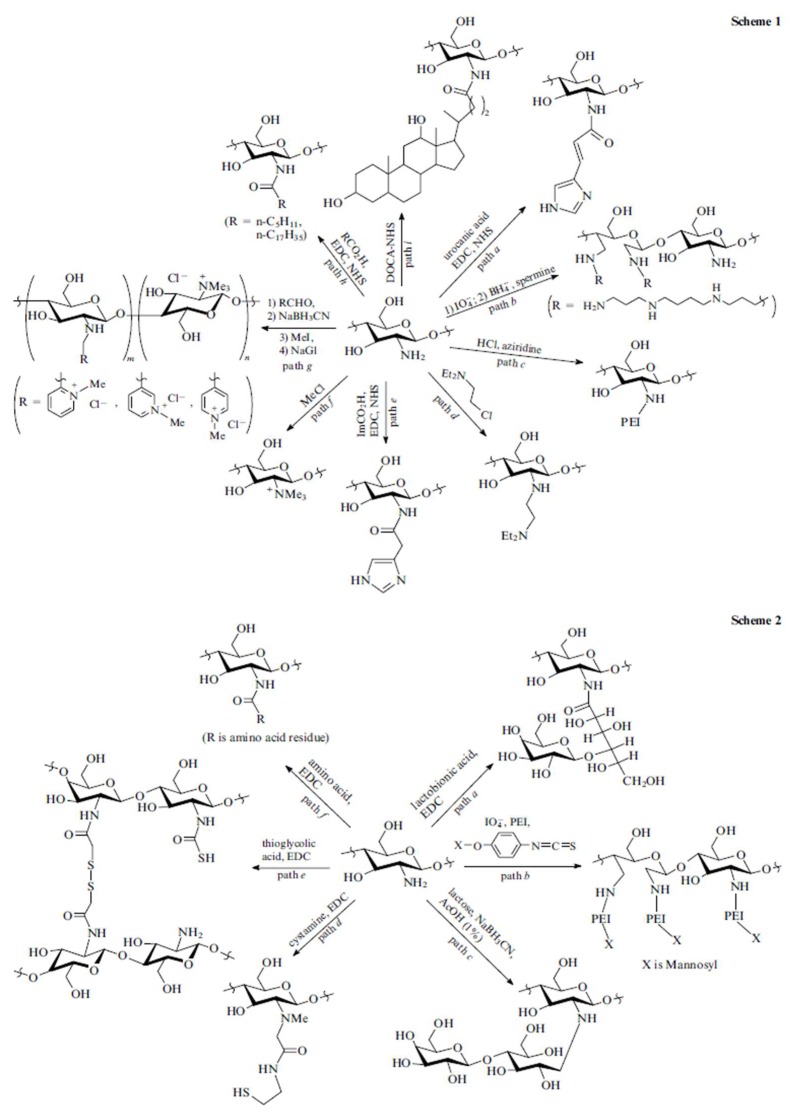
Scheme 1: hydrophilic modification to chitosan; Scheme 2: hydrophobic modification to chitosan. EDC: 1-ethyl-3-(3-dimethylaminopropyl) carbodiimide, NHS: N-hydroxysuccinimide, DOCA-NHS: NHS ester of deoxycholic acid, ImCO2H: imidazo-4-ylacetic acid. [14] (Reproduced with the permission of Turpion-Moscow).

Although chitosan offers several favourable characteristics as an excellent gene carrier, unmodified chitosan is restricted by its low solubility in physiological conditions. Therefore, chitosan derivatives with enhanced solubility compared to chitosan for gene delivery have been well explored and summarized in several reviews [24,25,26]. For example, Andrey S. et al. [14] have explored the covalent modification of chitosan by hydrophilic modification (Scheme 1 in Figure 2) or hydrophobic modification (Scheme 2 in Figure 2). The main advantage of hydrophilic modification to chitosan is to increase its solubility. One of the most popular approaches for chitosan hydrophilic modification is to conjugate poly (ethylene glycol) (PEG) to chitosan. Gutoaia et al. [27] reportedly applied different amounts of PEGylation to chitosan and characterized the properties of the resultant PEGylated chitosan nanoparticles. They found that PEGylation did not affect the binding capacity of siRNA. Furthermore, higher degree of PEGylation could enhance chitosan solubility, reduce nanoparticle size and zeta-potential of the nanocomplexes. However, PEGylation of chitosan caused lower cellular uptake and decreased transfection efficiency due to several reasons, such as the inhibition of cellular uptake (repulsion from PEG) and poor endosomal escape efficiency. Therefore, it is critical to fine-tune PEGylation degrees to generate chitosan/siRNA nanocomplexes with maximum transfection efficiency. In addition to PEG, some other hydrophilic modifications have also been conjugated to chitosan for gene delivery, such as dextran, poly (vinyl pyrrolidone), poly (β-malic acid) and poly (aspartic acid) [25]. Most of the covalent conjugations are based on the modification of amine groups of chitosan, which may decrease the charge and finally affect the gene binding efficiency. 

The introduction of hydrophobic modification to chitosan offers several advantages, such as easy binding to cells, enhanced nanoparticle stability in serum, better cellular uptake and protection from degradation, and facilitating DNA dissociation from chitosan inside cells [14,28]. For example, hydrophobic stearic acid (SA) was conjugated to chitosan through EDC/NHS method (path f, Scheme 2 in Figure 2) to form amphiphilic polymer. These self-assembly SA-chitosan micelles showed low cell toxicity and high transfection efficiency [29]. Spermine-grafted SA-chitosan was explored for pDNA delivery. These spermine-SA-chitosan/pDNA nanocomplexes exhibited high DNA binding ability, lysosomal escape property, high gene knock-down and low cytotoxicity [30]. More chemical modification methods of chitosan can be found in reviews done by Hu-lin Jiang et al. [25] and Andrey et al. [14]. Although several chitosan derivatives have been developed, continued preclinical studies are necessary to fully understand their safety for use and efficiency. 

### 2.5. Chitosan Deacetylation Degree

The degree of deacetylation (DD) determines the positive charge and solubility of chitosan, the binding capacity of gene, cellular uptake and finally the transfection efficiency [16,31]. Higher DD translates to more primary amines in a chitosan molecule, increased positive charge and eventually higher capacity of gene complexation. Koping-Honggard et al. [32] reported that chitosan DD must be more than 65% to achieve stable complexes with pDNA. Higher DD (≥80%) of chitosan is mandatory to complex siRNA and form stable nanoparticles due to siRNA’s highly negative charge [33]. Malmo et al. [22] found that a 100% deacetylated chitosan was more efficient in gene silencing than partially deacetylated chitosan. This was because, fully deacetylated chitosan possessed more amine groups (higher positive charge) and thus, could more effectively escape from endosomal/lysosomal compartments.

### 2.6. N/P Ratio

N/P ratio is defined as the ratio between chitosan nitrogen (N) per gene phosphate (P). The surface charge of polyplexes depends on the mixing molar stoichiometry of gene to chitosan (N/P ratio). N/P ratio is a critical factor that affects polyplex stability, the interaction with cells and ultimately determines the transfection efficiency. A higher N/P ratio means a higher chitosan concentration in the complex, which not only improves the stability of the polyplex but also enhances the interaction with cells resulting in better transfection efficiency. However, a very high N/P ratio complex may exhibit too tight a binding of the genetic material (too high a stability), and eventually reduce the release of gene from the complex and thus reduce the transfection efficiency. In contrast, a very low N/P ratio will yield neutral or negatively charged complexes, which will aggregate due to the absence of interparticle repulsive forces, affecting cellular internalisation and result in poor transfection efficiencies. Several studies reported that chitosan nanoparticles with N/P ratio >25 and DD of 80%~85% exhibited many issues during in vivo studies including blood incompatibility, limited release dose of the gene and non-specific effects due to large quantities of free excess cationic chitosan [16,22,34]. Table 1 summarizes the different chitosan formulations, listing chitosan molecular weight, chitosan derivatives, deacetylation degree, N/P ratio, size, cell type and transfection efficiency.

### 2.7. Chitosan’s Toxicity

Chitosan, a naturally occurring polysaccharide, is generally regarded as a non-toxic and biocompatible polymer. It is used as food additive in Japan, Italy and Finland and is also listed as a GRAS (Generally Recognized As Safe) product in the U.S. [26,43]. Furthermore, chitosan has been employed in various FDA approved medical devices such as hemostatic dressing, bandages and a coating agent for contact lens [44]. However, there is still no chitosan drug delivery products approved in the market despite intensive research activities employing chitosan as drug delivery carrier. 

Although unmodified chitosan is generally non-toxic, the modification made to chitosan could potentially alter its safety profile and each modification should be assessed thoughtfully. Till now, most studies show that the toxicity of chitosan is dependent of molecular weight, degree of deacetylation and other factors like extent of quaternization [45]. A summary of the reported median lethal dose (LD_50_) and half maximal inhibitory (IC_50_) of chitosan and its derivatives is presented in a recent review by Thanou et al [43] and the IC_50_ is approximately 0.2–2.5 mg/mL in most cell models. For instance, in the case of chitosan/plasmid DNA nanoparticles, the pharmacokinetic and biodistribution profiles of the formulation are initially controlled by the nanoparticles properties such as size and charges. Further, cellular uptake kinetics may be altered due to quaternization of chitosan. Thanou et al. [46] investigated the effect of increasing quaternization of oligomeric/polymeric chitosans and therefore, positive charge on cell viability and transfection of monkey kidney fibroblasts (COS-7) and epithelial breast cancer (MCF-7) cells. It was found that all derivatives were significantly less toxic than linear polyethylenimine (PEI) and increasing toxicity was observed with increasing degree of trimethylation. Moreover, higher toxicity was seen in polymeric chitosan derivatives over oligomeric chitosan derivatives at similar degree of trimethylation. Similar observation was made by Zubareva et al. [47] that quaternization of chitosan led to the increase in cell penetration and cytotoxicity manifested by reactive oxygen species production, cell cycle arrest and inhibition of cell proliferation.

When it comes to in vivo toxicity, current studies appear that chitosan shows minimal toxic effects [14,42]. In the vertebrate bodies, chitosan is mainly hydrolyzed enzymatically by lysozymes and chitinases like glucosamine-glucosamine, glucosamine-N-acetyl glucosamine and N-acetyl glucosamine-N-acetyl glucosamine [44]. Several studies on the in vivo biodistribution of chitosan nanoparticles showed that the biodistribution is controllable through route of administration and both molecular weight and formulation dependent [43,48]. In term of intravenous administration, the main organ of uptake is the liver and accumulation was found to increase with increasing molecular weight [48]. In term of oral administration, Piyasi et al. [49] assessed the in vivo chronic toxicity of chitosan and its derivatives used as oral insulin carriers in a mouse model. It showed no significant adverse effects on the blood serum, liver and intestine of the treated animals and intestinal luminal bacteria were able to biodegrade the chitosan completely. Further, oral administration of chitosan (up to 6.75 g/day) to healthy human volunteers demonstrated its safety and the absence of side effects [50]. From the current studies it could be surmised that chitosan-based formulations exhibit low toxicity; however, care must be taken to ensure that its derivatives should assessed individually.

## 3. Preparation Methods of Chitosan Micro/Nano-Particles

**Figure 3:**
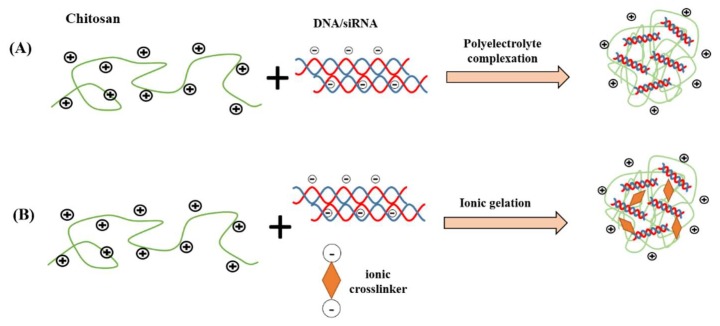
Common chitosan nanocarrier fabrication methods for nucleic acid delivery. (**A**) polyelectrolyte complexation; (**B**) ionic gelation.

Broadly, polyelectrolyte complexation and ionic gelation are the two most common techniques utilized to fabricate chitosan nanoparticles for gene delivery, as shown in Figure 3. The particle size, stability, transfection efficiency, toxicity, and the kinetic of the drug-release profile are the factors to be considered during selection of the fabrication technique. In the method of polyelectrolyte complexation, the protonated amines in the chitosan backbone enable positively charged chitosan to bind efficiently to negatively charged nucleic acid, leading to spontaneous formation of nanoscale polyelectrolyte complex (polyplex), which protects condensed nucleic acid from enzymatic degradation. During the fabrication, a gentle mixing of diluted chitosan and nucleic acid solutions followed by incubation typically generate chitosan/DNA particulate complexes driven by strong electrostatic interactions [28,51]. The properties of the polyplexes are significantly influenced by factors such as structural properties and concentration of chitosan and its derivatives, mixing regime and pH value [22]. In a study by Bozkir et al. [52], the team fabricated chitosan-plasmid DNA nanoparticles in the size range of 450-820 nm depending on the molecular weight of the chitosan used and found that the encapsulation efficiency of pDNA is inversely proportional with the molecular weight of chitosan. 

Generally, the pH of the chitosan solution during the nanoparticle fabrication is kept at a pH of 5.6–6.5 and alteration of the pH condition would have an impact on its physicochemical properties of the polyplexes. At acidic pH, below the pKa (~6.5), the primary amines of chitosan are highly protonated and become positively charged, facilitating nucleic acid binding and complex stability [26]. Under neutral or alkaline conditions, the degree of protonation is reduced and the electrostatic interaction between chitosan and nucleic acid becomes weaker, resulting in complex instability and dissociation of DNA/siRNA from the polyplexes. Studies had also showed that secondary (non-electrostatic) interactions, such as hydrogen bonding and hydrophobic interactions, could be responsible for the binding between chitosan and DNA under the neutral or alkaline conditions [33]. It should be worth noting that while the chitosan/nucleic acid nanoparticles formed at acidic pH is regarded as a stable complex, the stability and thus the transfection efficiency of the chitosan polyplex would be affected in vivo at the physiological condition. 

In particular, stable complexes are formed only when chitosan is added in molar excess relative to nucleic acid at certain N/P ratios. The N/P ratio is a critical factor in complex formation determining the overall surface charge of a polyplex, DNA-condensing ability and eventually transfection efficiency [26]. For instance [28], it was observed by atomic force microscopy that insufficient complex formation was observed at N/P ratio of 1 while spherical particles with diameter of 180 nm were formed at N/P ratio of 5. As noted above, a high N/P ratio leads to strong electrostatic interaction for DNA/siRNA condensing and protection, forming too stable a polyplex that does not release DNA/siRNA once the polyplex arrives at the site of action. Therefore, a good balance needs to be achieved by adjusting formulation-related parameters. While polyelectrolyte complexation is a simple method for chitosan nanoparticle formation with typically high encapsulation efficiency of nucleic acid, this method suffers from several limitations like: (i) dissociation of the complexes in the presence of polyanions, (ii) long term storage issue, (iii) undefined physical shapes, and (iv) limited capacity to co-associate other functional molecules, such as proteins, to the polyplex structure [45].

Alternatively, chitosan/nucleic acid nanoparticles could be fabricated using an ionic gelation method. In ionic gelation, the nucleic acid is entrapped within the chitosan matrix, which is physically crosslinked with ionic crosslinker agent, rather than fully depending on electrostatic interactions between chitosan and nucleic acid [53]. The ionic crosslinker agents are typically anionic in nature that strongly bind to chitosan, such as tripolyphosphate (TPP), thiamine pyrophosphate, sodium sulfate, dextran sulfate, poly-γ-glutamic acid and hyaluronic acid [54,55]. The use of ionic crosslinker in the ionic gelation method could increase the “hardness” of the nanoparticles, enhance the nanoparticles’ stability and further extend the drug release duration of the entrapped nucleic acids. Furthermore, this method provides the possibility to co-encapsulate protein or macromolecules that could confer additional advantages to the nanoparticles such as improving cell internalization or the intracellular trafficking of the DNA. For instance, Sharma et al. formed siRNA encapsulated nanoparticles via ionic gelation method by mixing TPP solution with siRNA prior to nanoparticle formation and this is added dropwise to the chitosan solution with constant stirring [56]. Positively charged spherical nanoparticles were produced with mean diameters less than 150 nm at all N/P ratios (20:1, 60:1 and 100:1), with high siRNA encapsulation efficiency (>96%). Complete binding of siRNA to chitosan nanoparticles was observed when the N/P ratio was 100:1 by gel electrophoresis. 

In another study by Csaba et al. [45], ionically crosslinked nanoparticles based on various molecular weight chitosans were formulated with plasmid DNA or dsDNA oligomers using the ionic gelation method with pentasodium tripolyphospate as the crosslinking agent. Low molecular weight chitosan nanoparticles gave high gene expression levels in HEK 293 cells two days after transfection, reaching a plateau of sustained and high gene expression between four and 10 days. Moreover, this method allows the inclusion of BSA macromolecule into the nanoparticles without altering the inherent transfection efficiency of the nanoparticles. In addition, DNA/siRNA can be encapsulated in chitosan by using conventional methods such as layer-by-layer coating for nanoparticle preparation [33,57,58].

## 4. Chitosan Based Formulations for siRNA/DNA Delivery

### 4.1. Formulations with Enhanced Stability

As mentioned earlier, the ideal siRNA/DNA vector should exhibit good stability in the biological environment. When administered intravenously, the immediate dilution of the formulation in blood components can extensively destabilize the delivery vehicle [59]. Especially for cationic chitosan-based formulations, polyanions in the blood (such as serum proteins and glycosaminoglycans) can compete with siRNA/DNA for binding, resulting in the unpacking of the chitosan-based complexes and lead to premature siRNA/DNA release before reaching the intended site [60,61,62]. Furthermore, the strong interaction of cationic chitosan formulations with anionic serum proteins (such as albumin or fibrinogen) could result in aggregate formation, rapid clearance, and cellular uptake inhibition, leading to embolism and increased toxicity [60,63].

To evaluate the stability of carriers in vitro, vectors are generally incubated with physiologically relevant medium under physiological conditions. The hydrodynamic sizes of the vectors are then monitored against incubation time. It was reported in a study that chitosan/DNA polyplexes (about 100 nm diameter) aggregated to micrometer-sized particles after 2 h incubation in PBS [64]. There are many other assays to determine particle stability. An example is the BSA challenge. Generally higher BSA concentrations will induce greater particle instabilities. For instance in one study, the group incubated their chitosan/DNA polyplexes with various concentrations of BSA, followed by measuring the alteration in absorbance at 350 nm [65]. They found a significant increase in absorbance for their formulations after 1-hour incubation with 2 mg/mL of BSA, indicating extensive particle instability [66]. 

Regardless of the possible issues mentioned above that could impact the effectiveness of chitosan-based particles, it was found that chitosan does impart stability to siRNA in the presence of serum. For instance, stability studies revealed that chitosan nanoparticles were stable in medium containing up to 10% serum for chitosan–siRNA complexes and chitosan–TPP–siRNA nanoparticles [67]. Katas et al. incubated free and chitosan complexed siRNA in 5% FBS serum at 37 °C and found that free siRNA started to degrade after 30 min (fully degraded after 48 hour). In contrast, siRNA recovered from chitosan–TPP nanoparticles started to degrade only after 24-h incubation (fully degraded after 72 h). The experiment was further repeated by incubating siRNA as well as chitosan–siRNA nanoparticles in a 50% serum concentration. Immediate degradation of siRNA was observed upon mixing with serum. In contrast, the siRNA recovered from chitosan–siRNA nanoparticles were intact up to 7 h (fully degraded after 48 h) incubation in 50% serum [67].

Various modifications have been done on chitosan to enhance the stability and circulation time of chitosan-based deliveries. Quaternization [46,68], glycosylation [64,68] and hydrophobic modifications are some of the modifications known to improve the stability of chitosan-based formulations [66,69]. The most common modification however, is the conjugation of PEG to increase polyplex colloidal stability and inhibit electrostatic interactions with negatively charged components of serum and cell membranes [70,71], achieved possibly due to the highly flexible PEG chains sterically interfering with nonspecific interactions between the serum-driven components and polyplexes [72]. PEGylation also provides stabilization by reducing inter-particular aggregation [73].

Studies have shown that PEGylating chitosan enhanced stability of chitosan deliveries in human plasma. For instance, H.Ragelle et al. [74,75] used fluorescence fluctuation spectroscopy [76] and single particle tracking (SPT) to investigate the stability of siRNA-chitosan carriers in human plasma. FFS is a microscopy-based technique that can monitor the integrity of the delivery system, while SPT allows the determination of the size distribution of the nanoparticles in human plasma. They reported that chitosan-siRNA formulations fabricated without PEGylated chitosan showed 70% of their initial amounts of siRNA released within 30 min in human plasma. The result was indicative that these formulations could have disassembled with plasma incubation and were not stable as siRNA carriers in vivo [77]. They explained that the instability could be attributed to the deprotonation of some of the chitosan amino groups at physiological pH, resulting in reduced binding to siRNA. Furthermore, the presence of anionic plasma proteins competes with siRNA for binding to chitosan, possibly resulting in the disassembly. PEGylated nanoparticles, in contrast, did not dissociate in human plasma at all after 2 h incubation, proving that using PEGylated chitosan for siRNA deliveries formulations enhances stability in plasma [56]. While PEGylation seems to indeed enhance the stability of the chitosan formulations, it is important to note that excessive PEGylation could mask the overall cationic charge of the carriers and cause steric hindrance, limiting cell penetration, another important criteria for an ideal gene therapy carrier.

### 4.2. Formulations with Enhanced Cell Penetration

After introduction of the siRNA chitosan-based carriers into the body, it is of paramount importance for the carriers to locate the target cells and gain entry as efficiently as possible due to the factors mentioned earlier, which could compromise the integrity of the carriers, thereby releasing the siRNA prematurely. Besides, the target sites for gene therapy are located within the cells, with DNA being delivered to the nucleus while the siRNA target is located within the cytoplasm [78]. 

Carriers without any cell-specific ligand conjugations lack cell specificity, relying mainly on their surface characteristics and adopting non-specific adsorptive endocytosis for cell entry [79]. Thus, it is evident that carriers with net positive surface charges, chitosan-based formulations for instance, could potentially achieve enhanced electrostatic surface binding on negatively charged cellular membranes, promoting good internalization [80,81,82]. Many research groups have reported that the size and shape of nanoparticles could largely affect cellular entry efficiency [33,83]. However, cellular internalization of nanoparticles in the presence of serum has been shown to not be affected by their starting size, due to opsonin proteins present in serum that could rapidly change nanoparticle morphologies and cause aggregation/precipitation [20,83]. To investigate cellular internalization, cells are commonly dosed with fluorescently labelled particles of interest and incubated over 4 to 6 h, followed by qualitative confocal microscopy imaging to visually observe particles locating within cells and quantitatively measured using flow cytometry to derive with the percentage of defined cell population with presence of particles of interest residing in cells. 

Chitosan carrier surfaces can be modified or decorated with ligands to enhance cellular entry and specificity according to the predominant types of protein receptors found on the target cells surfaces. In this way, cationic chitosan carriers can not only be electrostatically attracted to anionic cellular membranes, enabling ATP-driven cell penetration, but also enter cells via receptor mediated endocytosis with ligand-receptor interaction. Examples of ligands conjugated to chitosan formulations are transferrin [63,84], folate [85], mannose [86] and galactose [87,88,89]. Factors to consider when introducing ligands to carriers include the degree of ligand substitution, conjugation chemistry, spacer length between the ligand and polymer, and binding strength between the ligand and receptor [33,90]. Cell penetrating peptides have also been utilised to enhance cell penetration of chitosan-based carriers. For instance, M. Malhotra et al. [91] developed TAT peptide-tagged PEGylated chitosan nanoparticles to deliver siRNA to neuronal cells.

### 4.3. Formulations with Enhanced DNA/siRNA Release

Following the successful entry of the siRNA carriers into the intended cells, the release of biologically active nucleic acids within the cells is equally important. Upon endocytosis, the carriers will be encapsulated within early endosomes, which will progressively mature into late endosomes with increasing acidification to pH 5–6 due to proton accumulation via an ATPase proton pump. Ultimately, late endosomes will fuse with lysosomes, forming an aggressive enzyme-rich environment of pH 4–5 [77]. Evidently, it is important to design carriers with the abilities to escape endosomes before the fusion with lysosomes. Chitosan based formulations could effectively escape endosomes, explained by their proton sponge effect mechanism. Chitosan with a pKa of about 6.5, will have its amine groups progressively becoming protonated with the increasingly acidified endosomal environment upon encapsulation of the carriers in endosomes. The heightened cationic charges will illicit an influx of water and chloride ions into the endosomes in attempt to neutralise the charges, resulting in extensive osmotic swelling and eventual physical rupture of the endosomes, releasing the chitosan carriers into the cytoplasm [77,90,92]. 

To enhance endosomal release, several agents have been included in various chitosan-based formulations. Some of these agents include fusogenic peptides [93,94] and pH-sensitive neutral lipids [95]. Fusogenic peptides undergo structural changes from a random coil structure at physiological pH to an amphiphilic α-helical structure at lower pH, which enhances endosomal membrane penetration. Neutral lipids, such as dioleoylphosphatidylethanolamine (DOPE), promote endosomal escape by enhancing fusion of carriers with the endosomal membrane.

### 4.4. Formulations with Enhanced siRNA Prolonged Gene Silencing

Depending on the transfection efficiency, construct and purpose of a DNA carrier, transfection could be transient or stable, with stable formulations incorporating DNA into the host cellular genome permanently. It is evident that carriers transporting DNA material need to penetrate into the nucleus for transfection. Vectors trafficking siRNA on the other hand, only need to transport and release siRNA into the cellular cytoplasm to inhibit the formation of specific proteins. Formulations transporting siRNA designed till date are generally transient in their gene silencing effect. As mentioned earlier, the arduous process of dodging opsonin proteins, penetrating target cells and escaping from endosomes, saw many formulations delivering just enough siRNA for transient gene silencing. 

To achieve a more prolonged gene silencing effect with siRNA, special design modifications have to be taken into consideration to develop a vector which can not only safely deliver adequate amounts of siRNA into the cellular cytoplasm, but also sustain release siRNA within the cytoplasm over the desired period of gene silencing. For instance, in attempt to protect the loss of alveolar bone in periodontal diseases, Z. Ma et al. applied [96] an RNAi-based therapeutic strategy to silence *RANK* signaling using thermosensitive chitosan hydrogel as siRNA reservoir and vector. They reported that RAW264.7 cells treated with hydrogel loaded with si*R-RANK* demonstrated a significant prolonged *RANK* mRNA knockdown effect of 30% (three days), 50% (six days) and 60% (nine days) post transfection. However, protein quantification assay was not conducted to affirm ultimate protein down regulation. Furthermore, in vivo release studies saw the formulation having a significant siRNA burst release of 80% 1.5 days post treatment to mice.

Another study from the same group that reported sustained gene silencing with their formulation was a study by W. Song et al. to surface-modify titanium to improve bioactivity and bone binding ability for bone implant materials [57]. To functionalize titanium with siRNA for sustained gene silencing in nearby cells, they made use of the layer-by-layer (LbL) approach. Multilayers of sodium hyaluronate (polyanion) nanoparticles and chitosan/siRNA (polycation) nanoparticles were built up forming a film on smooth titanium surfaces. To observe prolonged gene silencing, films fabricated with layers of hyaluronate and chitosan/si*GFP* were seeded with *GFP*-positive H1299 cells and fluorescence was quantified with flow cytometry after 3, 5 and 7 days. In vitro transfection revealed that the LbL film-associated siRNA could consistently suppress GFP expression in H1299 without showing significant cytotoxicity [57]. However, no qPCR or Western blot assays were conducted to affirm gene silencing nor was any animal work done to investigate effectiveness in vivo.

The works discussed showed promising potential in the development of chitosan-based siRNA deliveries for prolonged gene silencing. More work should be done in this area to develop siRNA formulations with prolonged gene silencing abilities which can be utilised in disease models requiring extended periods of inhibitions of undesirable proteins.

### 4.5. Recent Enhanced siRNA/DNA Delivery Systems

Based on the various enhancements mentioned earlier, many research groups have successfully devised different siRNA/DNA therapeutics for many disease models (Table 2). Formulations in the past usually showed good gene silencing/transfection effects in vitro but limiting effect in vivo due to factors mentioned earlier. With recent understanding of the drawbacks of chitosan, various research groups have developed and modified their formulations by improving chitosan solubility, targetability, buffering capability, and unpacking ability [24]. Thus, some working deliveries can be seen showing promising gene silencing/transfection effects in vivo in recent works. Although extensive research has been done on chitosan-based gene delivery vectors, clinical translation of chitosan-based delivery system has not been reported yet. The development of hybrid chitosan-based vectors may overcome current drawbacks and bring new possibilities to non-viral vectors. For example, Kang et al. [97] prepared Au nanoparticle (core)/ siRNA first, and then conjugated glycol chitosan/taurocholic acid to the surface of Au-siRNA. They found that chitosan-taurocholic acid protected siRNA against degradation in GI, facilitated active transport to liver, enhanced selective accumulation in colorectal liver metastases and successfully initiated cancer cell apoptosis. 

Several research groups are focusing on prolonged in vivo gene silencing/transfection effects with their siRNA/DNA formulations. Customized siRNA/DNA delivery with gene silencing/transfection durations and siRNA/DNA combinations introduced together, catered to different disease models could be expected in time. Specifically, for gene silencing, our group has developed a cell-mimicking nanogel approach. Chitosan was methacrylated to form UV crosslinkable chitosan-methacrylate (MA) monomer, which can not only form a complex with siRNA but also increase the solubility of chitosan. The lipid thin film was hydrated with siRNA, chitosan-MA and photo initiator. After extrusion and 365 nm UV photopolymerisation, chitosan-methacrylate based nanolipogel (NLG) was fabricated. The encapsulation efficiency of siRNA was about 85% and NLG was able to sustained-release siRNA up to 42 days in vitro. After cellular internalisation, covalently crosslinked chitosan NLG networks can control release of siRNA in the cytoplasm, overcoming the drawbacks (poor stability and lack of sustained-release) of traditional chitosan nanoparticles. Moreover, the bilayer of siRNA encapsulated chitosan NLG can be easily modified to target specific cells or tissues.

## 5. Outlook

The success of a gene therapy strongly depends on the gene delivery systems, which should possess the following characteristics: targeting of the specific cells, low cytotoxicity, high gene delivery capacity, protection of gene cargo, sustained-release of the gene, escape capability from lysosome, and high transfection efficiency. Chitosan-based gene carriers have high therapeutic potential because they meet most of the required characteristics. However, some limitations remain: its low solubility at physiological pH, the stability of the complexes after cellular uptake, and premature release inside the cytoplasm. Some strategies to improve the performance of chitosan-based carriers are emerging. For example, chemical modification (PEGylation) of the chitosan has been used frequently to improve the solubility of chitosan. However, excessive modification may reduce the positive charge density of chitosan and affect its binding capacity to genes. Chemical modifications to siRNA can also increase the stability of nanoparticles but it often renders siRNA ineffective. Another approach is the addition of negatively charged components to stabilize the chitosan nanoparticles. 

As mentioned in Section 4.5, we have designed a cell-mimicking NLG nanoparticle for siRNA delivery that mimics the transport-controlling components of a cell (membrane and liquid-gel like cytoplasm). With this approach, we could overcome some of the limitations mentioned above, particularly for gene silencing applications. The high encapsulation efficiency of bioactive agents and its sustained release capability after entering cells, are huge advantages over existing systems, that we plan to explore further in animal models of specific diseases, including unwanted fibrosis. More work and other approaches to modifying chitosan are needed to fully exploit the unique nature of chitosan.

## Figures and Tables

**Table 1:** Typical examples of chitosan-based carriers for gene delivery.

Chitosan MW (kDa)	Genes	Chitosan Type	DD (%)	N/P Ratios	Polyplex Size [32]	Cell Type	Transfection Efficiency (%)	References
10	siRNA	Chitosan	80, 92	5, 10	78–235	HepG2	56, 72	[35]
Medium MW(Sigma)	dsRNA	Chitosan	75	-	100–200	Sf21 cell line	85	[36]
Low MW(Sigma)	siRNA	PEGylated chitosan/ Chitosan	-	-	100–150	HCT-116	72/69	[37]
400	siRNA	Chitosan (with dextran)	87.3	-	111–172	HCT-116	-	[38]
5~15	siRNA	Chitosan	84	30	108	HEK293	3.9	[39]
150~400		Chitosan	86		187		12.3	
42~50		Trimethyl chitosan	84		138–162		24.3–37.1	
7.5-11.3	pDNA	Chitosan	92–94	-	80–100	HEK293	>65	[40]
Low MW	pDNA	Chitosan	90	5,7.5,10	300–1000	A549	-	[41]
470	siRNA	Chitosan glutamate	86	-	311–350	Jurkat, A3.01	90	[42]

**Table 2:** Representative examples of chitosan-based carriers mediated gene delivery.

**Disease Model/Goal**	**siRNA Formulation**	**Target Gene**	**Protein/mRNA Silencing (%)**	**Cell Model**	**References**
Enhance bone binding ability in bone implant materials.	Multilayers of sodium hyaluronate and chitosan/siRNA nanoparticles on smooth titanium surfaces via Layer-by-Layer.	GFP, Ckip-1	Reduction of GFP at day 3, 5 and 7 by 70–80%. Osteogenic differentiation of MG63 increased with Ckip-1 silenced. Both in vitro.	MG63, H1299	[57]
Protect alveolar bone loss in periodontal diseases.	Thermosensitive chitosan hydrogel as siRNA reservoir and vector.	R-RANK	Reduction of mRNA at day 3 (30%), day 5 (50%) and day 7 (60%) in vitro.	RAW264.7	[95]
Promote nerve regeneration and local nanotherapeutics delivery.	Polymer filaments nerve implants loaded with chitosan/siRNA nanoparticles.	RhoA	Reduction of mRNA at day 2 (65–75%). Undisclosed reduction of protein at day 3.	PC12	[98]
Delivery of therapeutic molecules to the brain for the treatment of Neurodegenerative diseases.	Chemoselective conjugation of monomethoxy PEG, at the C2 hydroxyl group of chitosan polymer, with conjugation of PEG to a cell-penetrating peptide, Trans-Activator of Transcription.	Ataxin-1	Reduction of protein (100%) at day 2 in vitro.	Neuro2a	[91]
Efficient delivery of siRNA to the brain to combat Alzheimer's disease.	A peptide derived from rabies virus glycoprotein linked to siRNA/trimethylated chitosan through PEG.	BACE1	Reduction of protein (50–57%) after 2 days in vitro.	Neuro2a	[99]
Self-crosslinking nanoparticle to deliver polymerized siRNAs for tumour targeting cancer treatment.	Forming stable nanoparticles of thiolated glycol chitosan with poly-siRNA through charge–charge interactions and self-cross-linking simultaneously.	VEGF	Reduction of mRNA (95%) after 2 days in vitro. Reduction in mRNA (64%) after 24 days in vivo (particles injected every 3 days).	PC-3/tumour-bearing mice	[69]
Treatment of multi-drug resistance for tumour treatment.	Self-polymerized 5′-end thiol-modified siRNA incorporated to chitosan nanoparticles.	Pgp	Reduction of protein (62%) after 2 days in vitro. Reduction of protein (92%) after 22 days in vivo (particles injected every 4 days.)	MCF-7/ADR/MCF-7/ADR mice	[100]
Improving structural stability of siRNA for prolonged therapeutic efficacy.	Nanoparticles of siRNA/chitosan grafted with deoxycholates poly (D, L-lactic-co glycolic acid) (PLGA)	GFP	Reduction of GFP at 5 hour (60%) and 2 days (36%). Reduction of GFP at day 7 (undisclosed%) in vitro.	MDA-MB-435-GFP	[42]
**Disease Model/Goal**	**DNA Carrier Formulation**	**Target Gene**	**Protein/mRNA Expression**	**Cell Model**	**References**
Enhanced DNA delivery vehicle to efficiently transfect cells under physiological conditions.	Liposome (DPPC, cholesterol) encapsulated chitosan/DNA nanoparticles.	Luciferase/ GFP	Luciferase expression observed in vitro. GFP expression observed in vivo.	HEK 293/ chorioallantic membrane model	[101]
DNA delivery nanoparticle to efficiently transfect intraocular retinal cells under physiological conditions.	Non-viral nanoparticles composed of glycol chitosan and plasmid DNA.	CBA-eGFP	GFP expression observed in vivo at day 14 post-injection in the retinal pigment epithelium.	Adult wild-type albino mice (eyes).	[102]
Examine effectiveness of dry gene powders for treating lung metastasis.	Dry chitosan-DNA powders prepared by dispersing a chitosan–DNA solution into supercritical carbon dioxide	Luciferase/ GFP/Muβ	Luciferase and GFP expressions observed. Lung metastasis suppressed at day 21.	CT26/mice with CT26	[103]
Enhanced stability and efficacy of DNA delivery vehicle.	Chitosan/DNA complex formulations with chitosan of varying molecular weights.	GFP	GFP expression observed (13 days). Reverse transfection showed 150% increased efficiency compared to standard protocol.	HEK 293	[103]
Development of gene-activated collagen scaffold with chitosan/DNA nanoparticles for tissue engineering.	Oligomeric chitosan with DNA complexation followed by further crosslinking with TPP. Particles are soak-loaded onto hydrated collagen scaffold.	Luciferase/GFP	Luciferase expression showed overall transgene expression. GFP expression sustained over 14 days.	mesenchymal stem cells (MSC)	[104]
Using efficient chitosan/DNA activated scaffolds to accelerate bone regeneration in critical-sized bone defects.	Chitosan/DNA complex soak loaded into fabricated collagen scaffold.	BMP-2/VEGF/GFP	Both BMP-2 and VEGF expression observed in vitro over 14 days. GFP expression in in filtrating host cells at day 7 post in vivo implantation of scaffold.	mesenchymal stem cells (MSC)/calvaria of Wistar rats	[105]
